# Dichromated Gelatin in Optics

**DOI:** 10.3390/gels11040298

**Published:** 2025-04-17

**Authors:** Sergio Calixto, Mariana Alfaro-Gomez

**Affiliations:** 1 Centro de Investigaciones en Óptica, Loma del Bosque 115, León 37150, Mexico; 2 Departamento de Matemáticas y Física, Universidad Autónoma de Aguascalientes, Av. Universidad # 940, Ciudad Universitaria, Aguascalientes 20100, Mexico; mariana.alfaro@edu.uaa.mx

**Keywords:** photosensitive materials, dichromated gelatin, gelatin, holography, solar concentrators, DCG optical elements, humidity sensor

## Abstract

Dichromated Gelatin (DCG) was first used in optics in 1872 by Lord Rayleigh. Then, in 1968, Shankoff suggested its use as a photosensitive material to record interference diffraction gratings and holograms. Diffraction efficiencies of nearly 100% were achieved. This review discusses some physical and chemical characteristics of DCG films; the fabrication methods of DCG films; and some of the applications of DCG films in holography, holography in real time, solar concentrators, optical elements, and relative humidity sensors.

## 1. Introduction

Georg Adolf Suckow was born in Germany. He developed research, at the Heidelberg University, in physics, chemistry, and natural history and was the curator of the natural history museum in Mannheim. During his research, he worked with mixtures of dichromates with organic materials and other substances. He discovered the light sensitivity of these mixtures (1830) [1], in particular, the sensitivity of a mixture of gelatin with dichromates (DCG). Later in 1872, Lord Rayleigh [2] (J.W. Strut) mentioned this phrase in his article: “It will be remembered that a mixture of gelatin and bichromate of potash is sensitive of light, it becomes insoluble even in hot water, after exposure”. In addition, he reported the fabrication of gratings by the contact copy method using DCG. His fabricated diffraction gratings had a 120 lines/mm spatial frequency. This was the first optical element made with DCG. In 1968, Shankoff [3] used DCG to make volume gratings that presented about 98% diffraction efficiency. He used a development that comprised soaking the DCG plate in water and then drying the plate by soaking it in alcohol. With this method, a volume optical element was fabricated.

The relief or volume optical elements made with DCG could be present in lenses, zone plates, holograms, diffraction gratings, and more. The elements can be used in several applications, described clearly by Pawluczyk et al. [4]. As mentioned in the reference, these applications include “dispersive elements in spectroscopy and tunable lasers, pulse compressors or expanders in laser systems for ultrashort light pulse processing, as solar power concentrators, beam-splitters in interferometers, as scanners in bar code readers, as directional diffusers in image projectors and more”. These elements made with DCG show outstanding optical properties such as high transparency over the visible spectrum, low optical noise, low scattering, and absorption. Furthermore, the material bulk of the DCG plate could attain large refractive index modulation and high resolution. Another good characteristic is its re-processibility [5].

Because this article will appear in the issue named “Design and Development of gelatin-based materials”, this Review should be taken as an introduction to DCG in optics and is intended for people outside the optical discipline because it just mentions the main characteristics of the DCG film and some applications. The number of publications in optics involving the DCG is large, and it would be impossible to mention all of them here.

## 2. Physics and Chemistry of DCG

### 2.1. Colloids and Gelatin

**Figure 1:**
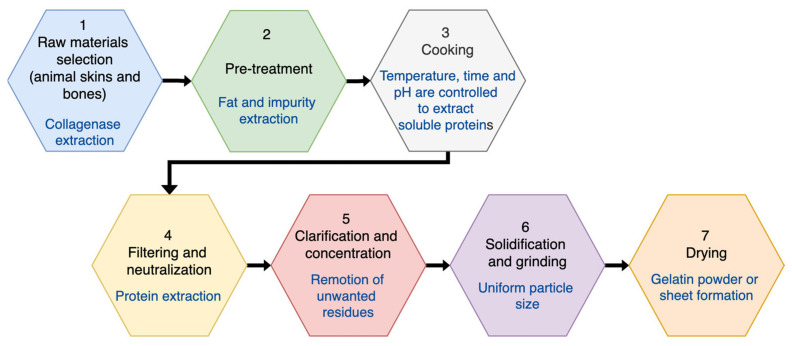
Gelatin fabrication process.

Gelatin is manufactured from the protein collagen [1,6] (Figure 1). The primary collagen sources for gelatin are cattle hides and bones, but pig and fish skins are used as well. Bones have a large mineral content of hydroxyapatite that is removed. The properties of gelatin are connected with details of its native protein precursor. Collagen has the role of forming the network of connective tissues such as skin, bone, cartilage, tendon, and ligaments. Collagen has a molecular length of about 2850 Å with a diameter of 14 Å; that is, it has a rod-like shape. The collagen structures present a right-handed triple helical helix to give a demineralized bone. Both cattle hide and ossein (organic extracellular matrix of bone, which is made of 95% collagen) have a prolonged pretreatment with dilute alkali at pH 12, at ambient temperature over months. The extracted gelatin is subsequently adjusted to a desired pH of about 5–6 and clarified by filtration. Enzyme-assisted extraction of gelatin from collagen has been attempted, but it was not successful. The properties specified for gelatin are usually viscosity, setting time, and rigidity, which depend on the molecular size, degree of chain branching, and the chemical nature. Differences in chemical composition among gelatins or the different chains of gelatin are a potential source of variations in certain bulk physical properties. The tendency for water to enter and swell a gelatin layer will be governed by the thermodynamically favored dilution effect on the concentrated network of polymer chains, internal and external osmotic effects, and viscoelastic effects, which depend on the crosslinking. Swelling also depends on pH. The overall manufacture process of gelatin is schematized in Figure 1 [7].

### 2.2. Gelatin Sensitizers: Ammonium, Sodium, and Potassium Dichromates

The sensitizing solution for dichromated gelatin layers consists of the organic colloid (gelatin) dissolved in a solvent (water). A mixture of dichromate dissolved in water is added to this solution. It is well known, since the 19th century, that potassium, sodium, and ammonium dichromates are sensitizers for gelatin. In particular, ammonium gives a high sensibility and presents high solubility in water [1,8]; therefore, this mixture allows the introduction of high concentrations when the DCG plates are immersed in the ammonium–water solution. When the sensitized DCG plates are illuminated, the hexavalent chromium along with the oxidable gelatin initiates the photochemical process. The Cr^6+^ ion is reduced to a lower ionization state: the trivalent Cr^3+^ ion, which forms a coordinated complex with the gelatin carboxylate group –COO-. This means there is a crosslink between the gelatin chains, presenting a high hardness in highly light exposed regions and a low hardness in unexposed regions. The hardening is enhanced and rapid when the films are dry because the molecules are closer to each other and when there is a high concentration. This hardening makes the films show more rigidity, decreases the solubility, and raises the melting point. As a result, the layer will swell less, thus taking less water.

All chromium compounds are classified as substances of significant hazard [9,10]. For example, trivalent and hexavalent chromium have been found to cause dermal irritancy, allergy, genotoxic effects, and carcinogenicity [10,11,12,13]. The poisonous effects over plants of potassium dichromate have also been studied [14], also indicating possible environmental hazards of the compounds [15].

### 2.3. Photochemical Processes Taking Place in the DCG Layer

Several attempts have been made to explain the formation of refractive index modulations by light in the DCG layer. Using X-ray fluorescence, the chromium content in the developed plate has been measured [16,17]. Two fabrication procedures were tested and are described in Section 3. In the first method, a solution of water + gelatin + dichromate is poured over a glass plate and left to dry. In the second method, a commercial holographic plate is fixed to take out the sensitive material, silver halogenates; afterward, the gelatin film is made sensitive by adding dichromate. After exposure to light from an argon laser, emitting at 514 nm, the plates were developed with a developer and then rinsed in water. X-ray fluorescence was used to analyze the plates, and it was found that, at the beginning, Cr(VI) was present in the DCG plate. Then, after exposure and development, the Cr(VI) was converted to Cr(III), which formed a crosslink bond (hardening) between the carboxylate groups of neighboring gelatin strands. That study showed that one chromium atom out of two was fixed in the gelatin structure.

### 2.4. Dark Reaction

When the recorded gratings are kept in the dark, without development, there exists a phenomenon called the dark reaction that will produce self-development resulting in a phase grating, and, with this, the diffraction efficiency will increase. Other non-desired characteristics that dark reaction gives are reductions in the shelf life and in the repeatability of the experimental results.

A characterization study about the dark self-enhancement of DCG plates under certain parameters was mentioned in reference [5]. After exposure, the diffraction efficiency continued to rise due to the dependence on the pH at the film fabrication time. For example, the diffraction efficiency was measured after the exposure time in an elapsed time from 0 days till 180 days later. Two DCG plates were considered; they were made with mixtures having pH values of (a) 6 and (b) 8, respectively. After 50 days, plate (a) showed a diffraction efficiency of 1% and plate (b) 1.6%. Then, after 180 days, plate (a) showed a diffraction efficiency of 1.7% and plate (b) a diffraction efficiency of 2.5%. More DCG plates’ behaviors considering the dark reaction are shown in reference [5]; for example, the diffraction efficiency as a function of pH, having as a parameter the days after the exposure time, when the plates were in a light-tight container.

### 2.5. DCG Films’ Spectral Sensitivity

The production of a chemical change by actinic radiation requires the absorption of this radiation. In dichromated colloid layers, the dichromates efficiently absorb ultraviolet, violet, and blue radiation; the maximum absorption by ammonium and potassium dichromates is at 357 nm and 367 nm, respectively [1]. Based on these spectral sensitivities, it is common to use lasers that emit UV, violet, blue, and green light. He-Ne lasers emitting at 632.8 nm cannot be used unless they are used in a method devised by Kubota [18] that sensitizes the plates to this wavelength. Holograms have been recorded mainly with the He-Cd, 441 nm, and the Argon ion laser (488 nm, 514.5 nm).

## 3. DCG Film Fabrication and Processing—Recording Gratings

**Figure 2:**
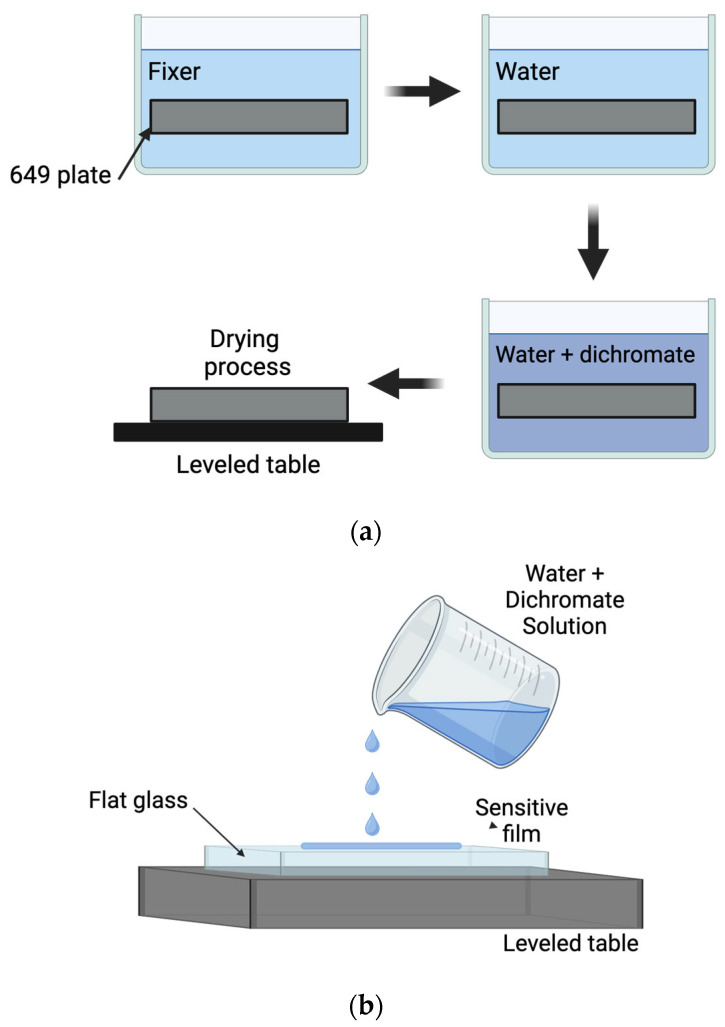

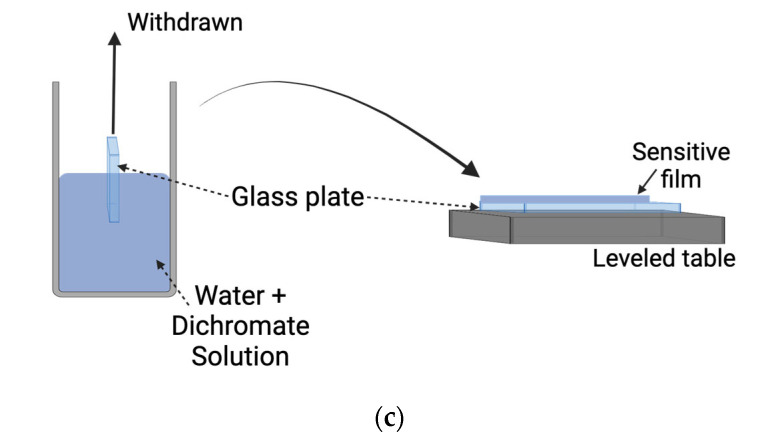
DCG film fabrication methods. (**a**) Plate (649 F) is fixed and then washed with water to obtain a thin gelatin film. (**b**) A clean flat glass plate is placed over a leveled table. A solution of gelatin and dichromate is poured over the plate and left to dry. (**c**) Dip-coating method. Figures created with BioRender.com, accessed on 21 March 2025.

There are three basic methods to make DCG plates (Figure 2). In the first one [8], a photographic plate is used (Figure 2a). At the beginning, the plate (649 F) is fixed and then washed with water. After this procedure, a thin gelatin film on a glass plate is obtained. Then, the plate is soaked in a water–dichromate solution and left to dry. In this way, a gelatin photosensitive layer is obtained. In the second method (Figure 2b), a clean flat glass plate is positioned over an acrylic table that has three screws that level the table; then, an amount of gelatin and dichromate solution is placed over the plate until it dries. The thickness of the sensitive layer is a function of the amount of the poured solution. In the third method (dip coating) (Figure 2c), a water solution of gelatin and dichromate is prepared in a beaker, and then a glass plate is dipped vertically in the beaker. It is vertically and slowly withdrawn with an instrument. One surface of the plate is cleaned, and then the plate is placed on a leveled surface until dry.

The first method presents a weakness because the photographic plates have been hardened at the factory. Thus, they could be too hard, and high exposures should be given. The second method is good because it is possible to make a layer with a given thickness, hardness, and sensitivity by using the right amount of dichromate. In the third method, only thin layers can be made.

**Figure 3:**
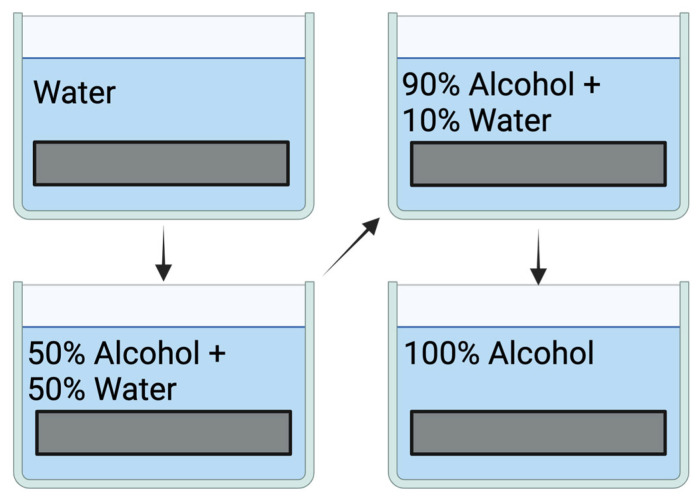
Development of DCG plates. Figures created with BioRender.com, accessed on 26 March 2025.

In the development step, there are mainly two processes [8]: (1) The exposed plate is immersed in water that will dilute the unexposed regions of the film; therefore, a surface relief will be shown. This process is traditionally used in photoengraving and in the fabrication of optical relief elements. (2) In the second process, the following steps are taken: (a) Wash in water for 10 min. (b) Soak in a mixture of 50% alcohol and 50% water and repeat in 90% alcohol and 10% water. Finally, soak in 100% alcohol (Figure 3). After this last development, the exposed and unexposed regions will present very different refractive indices in the bulk of the material, thus, giving phase holograms with high diffraction efficiencies.

One unique feature of holograms and relief optical elements made with DCG is that they can be reprocessed if they are erased by high humidity, for example, and the original optical element can be obtained [8]. It should be pointed out that the reprocessing procedure increases the noise level.

Once the DCG plates that were used to make reflection holograms were developed they presented diffraction efficiencies and spectral bandwidths. These values depend on the plates’ thickness and on the development process. Table 1 presents some typical values of these parameters.

Regarding the sensitivity of DCG films, there can be no unique measurement of sensitivity because this depends on the film thickness, grating period, recording wavelength, pre-hardening, developing processes, and others [8]. One of those sensitivity definitions is taken as the exposure when the diffraction efficiency presents a value of 20%. Table 2 shows some reported sensitivities where plates present different characteristics like thickness, grating periods, and developing processes. These values can be taken only as a guide.

To keep the scattering low after the developing process, a good gelatin bias hardness should be given in the sensitization step. Furthermore, the processing temperatures should not be high, and neutral pH values should be present in the baths.

**Figure 4:**
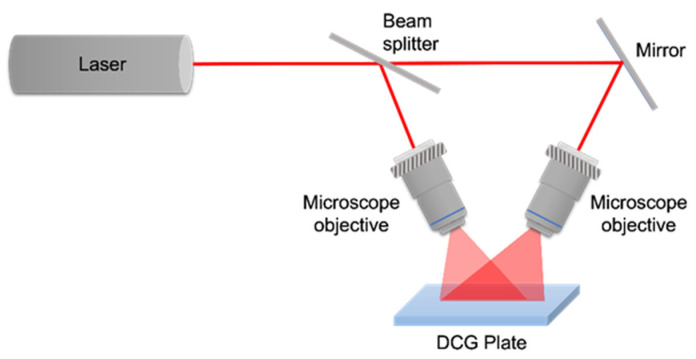
Recording configuration to make diffraction gratings.

A two-beam interference pattern, consisting of straight parallel lines, is recorded by a DCG plate for the fabrication (patterning) of diffraction gratings. Between the beams, there is an angle. If the angle is small, the spatial frequency of the interference lines is small, and the opposite, when the recording angle is big, the spatial frequency is high (Figure 4).

## 4. Special Developments of DCG Films

There are optical elements that present a relief. Thus, a study was developed and presented in ref [19] for structures recorded in DCG films. Because gelatin is mainly a protein, enzymes can be used to digest the gelatin. In the areas that are less exposed to hardening light, the enzyme will better digest the gelatin. However, in the areas that are more exposed to light, the enzyme will not digest too much of the gelatin. The surface’s height and shape are of interest because optical elements will show better efficiencies for given shapes and heights. In reference [19], it was found that the proteolytic enzyme papain, used in developing DCG, enhances the relief of the recorded structure. This result was obtained when gratings with a low spatial frequency were recorded: 8 lines/mm, 125 lines/mm spatial frequency. However, when higher frequencies were recorded, the results were not satisfactory. The action of the enzyme, at the microscopic scale, affects the gelatin layer in every direction, thus a dense structure, like the ones diffraction gratings with more than 125 lines/mm present, with a smooth surface will be disturbed and present a grainy structure and will finally be destroyed during the development process.

**Figure 5:**
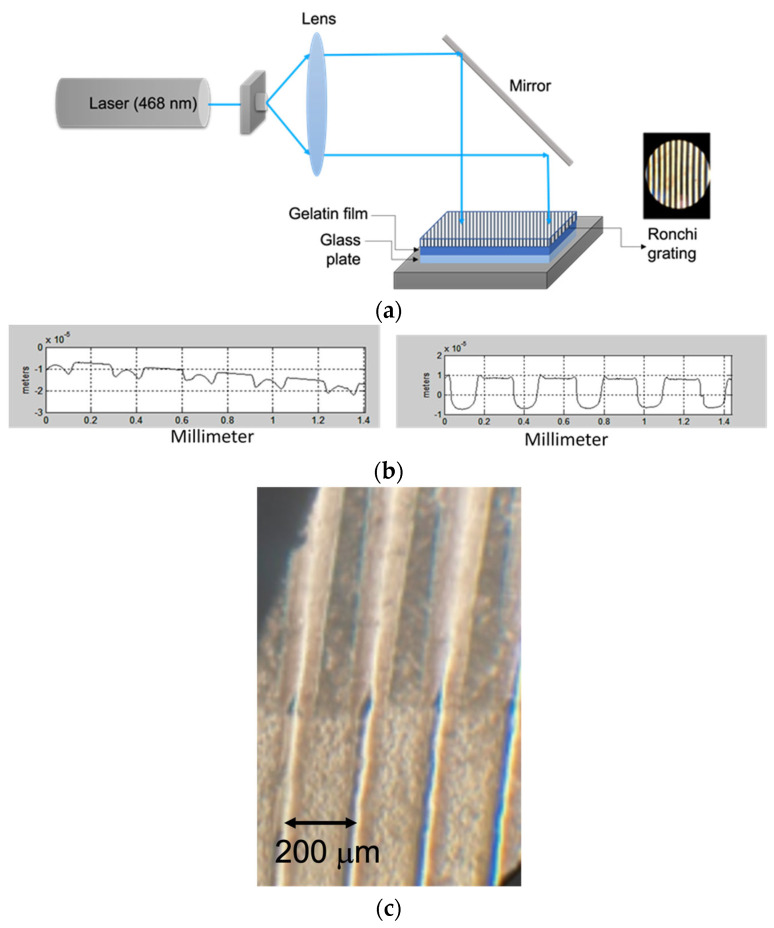
(**a**) Configuration to contact-copy a Ronchi grating on a DCG plate. (**b**) Profiles of two DCG gratings given by a profilometer. The one on the left suffered a development with just water, and the one on the right suffered a development with a mixture of papain and water. (**c**) Photograph of a grating that shows the development with water and papain in the upper section. The development with just water in the lower section.

In another study [20], lights with two recording wavelengths were used. One comprised a laser (λ = 468 nm), and the other used a mercury lamp that gave ultraviolet and visible light. In both methods, the contact-copy method, using Ronchi gratings, was used (Figure 5a). In optics, the exposure time and the development, when a surface relief element is made, should be considered. Profiles were measured with a profilometer. That study found that different grating surface reliefs were presented depending on the light source. A major result was that the DCG plates presented an increase in profile height when the papain development was used (Figure 5b). In Figure 5c, a portion of a grating is seen. The upper section shows the action of the papain–water mixture, and, in the lower section, just water was used.

## 5. Characterization of DCG Films

The DCG plates consist essentially of a glass plate, flat or curved, and a DCG film. The glass substrates can be inspected by interferometry [21] and should present optical quality. Regarding the gelatin film, it should present optical uniformity, which also can be tested by interferometry and by mechanical methods with a Talysurf, which can measure the surface irregularities with the help of a stylus. The stylus movement is converted into electric current changes (http://www.taylor-hobson.com, accessed on 14 March 2025). However, after the sensitization and developing processes, the gelatin surface suffers changes, and its thickness is increased. More film characteristics are shown in references [4,21] like the behavior of the refractive index in the sensitization process, the relation with relative humidity, the increase in the refractive index during exposure, and more.

Another characterization study, this time related with the DCG films’ reciprocity failure, was presented in reference [22]. The total exposure, E, is given by E = I × t, where I is the irradiance, and t is the exposure time. Given the exposure value for an ideal photosensitive material, the values of irradiance and time could change; however, the product should always be the same. However, this does not happen for all the possible values of intensity and time. This is called the “failure of the reciprocity”. This phenomenon is presented by the DCG plates and was tested by recording holographic transmission gratings with a two-step method using partially coherent light (wavelength of 405 nm). The light source was a high-pressure mercury lamp. The results were based on plots in which the diffraction efficiency was related to exposure and time.

## 6. Holography in Real Time with DCG Films

It has been mentioned in the paragraphs above that, after exposure, the DCG plates suffer a development process. However, a method has been mentioned in articles in which the plates can be used in real time; that is, no development is needed [23]. The recording wavelength mentioned was 514 nm, creating an absorption structure. At the same recording time, red light, 632 nm wavelength, was sent to the recording area. This red light is not absorbed by the plate, but it is diffracted by the recordings. However, if there is no development, the gratings present a low diffraction efficiency of about 1%. The characterization of the DCG plates was accomplished comprising the grating spatial frequency, exposure, and other parameters. Examples in the recognition of characters, enhancements of edges, image subtraction, two exposition holograms, and real-time phase conjugation were shown.

In another study [24], two interfering coherent beams generating a volume grating were recorded. Moreover, a theoretical study was developed considering the absorption of light by the sensitive gelatin layer. These experiments proved, as a main result, that there is a change in absorption during the exposure. Absorption increases when the exposure begins.

Another study of DCG plates, with self-development, used in real time was presented in references [25,26,27]. This time, glycerine was added to the DCG film. In a solution of water and dichromate at 6%, glycerin was added. Then, the plates were left in the refrigerator and later dried at room temperature. The film thickness was 400 µm. Recorded sinusoidal patterns were used to characterize the plates. A helium–cadmium laser (441 nm) was used as a light recording source. The two interfering beams made an angle of 14^0^; the intensities of both the recording beams were 4 mW/cm^2^, with the result that the maximum diffraction efficiency was 32%. However, when the recorded interference pattern increased the spatial frequency to 790 l/mm, 1180 l/mm, and 1330 l/mm, the diffraction efficiencies decrease to 21%, 6%, and 2%, respectively.

## 7. Solar Concentrators with DCG Films

**Figure 6:**
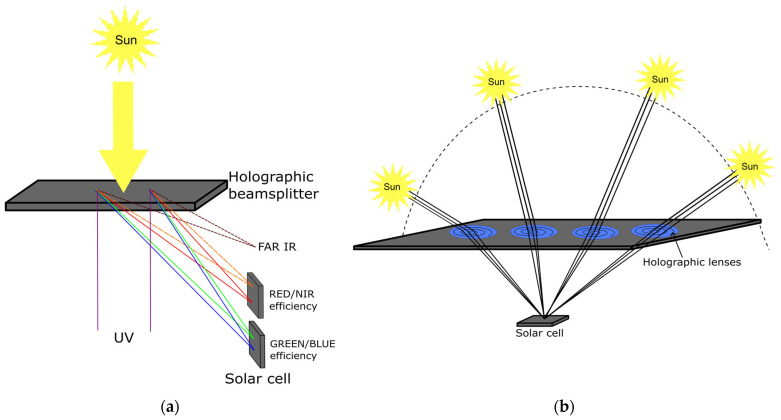
(**a**) DCG holographic structures allow the focusing of solar radiation over photovoltaic or thermal sensors. This optimizes the focusing of useful radiation over solar cells for different spectral bands and avoids heating by directing far-infrared wavelengths away from sensors. (**b**) The efficient concentration of solar energy can improve the cost–benefit ratio of using solar technology—photovoltaic or thermal—by creating structures that focus solar radiation at different day times, eliminating the need for expensive tracking systems.

The use of solar energy has become a very important area of research due to the possibility of using a clean and sustainable energy source. The use of solar energy to power up all human activities promises environmental benefits, decentralized power generation, and reduced energy costs, among others [28]. The efficient concentration of solar energy can improve the cost–benefit ratio of using solar technology—photovoltaic or thermal [29]. Holographic components have been proposed since 1987 [30] as a viable option for the concentration of solar energy [30,31,32,33,34]. Holographic lenses can eliminate the cost of tracking systems, simplify the concentrator design, and increase efficiency at defined wavelengths [35,36,37]. For the design and recording of an efficient holographic solar concentrator, the following aspects of the recording material must be considered: spectral sensitivity, dynamic and spatial frequency response, multiplexing capacity, toxicity, and time stability. The DCG shows good refractive index modulation and attains excellent diffraction efficiency and resolution, low noise, and good optical quality, which makes it suitable for solar energy transformation applications [38,39]. Different interferometric patterns can be recorded in DCG to create a holographic structure that diffracts light, allowing the focusing of solar radiation over photovoltaic or thermal sensors, as depicted in Figure 6 [37,40]. Given the high diffraction efficiency of DCG, volume holograms can be used to increase the conversion efficiencies [40]. DGC diffractive holograms can also be used to optimize the focusing of useful radiation over solar cells and avoid heating by directing far-infrared wavelengths away from sensors [41]. Although DCG holograms for solar concentration present many advantages, one should also consider the dichromate toxicity [42], low exposure sensitivity, and limited spectral response [5,30], which are the main disadvantages.

## 8. Control of Spectral Position and Bandwidth

As mentioned in the previous section, the possibility of designing holograms with a desired spectral pattern allows the optimal performance of DGC holograms for solar photovoltaic and thermal applications. The Bragg wavelength defines the spectral position, which refers to the most efficient diffractive wavelength of the hologram. The Bragg wavelength (*λ_B_*) depends on the fringe spacing *d* and the refractive index *n*, through the following relation:

(1) $$\lambda_{B}=2nd\sin\theta r,$$

where $\theta_{r}$ is the reconstruction angle.

**Figure 7:**
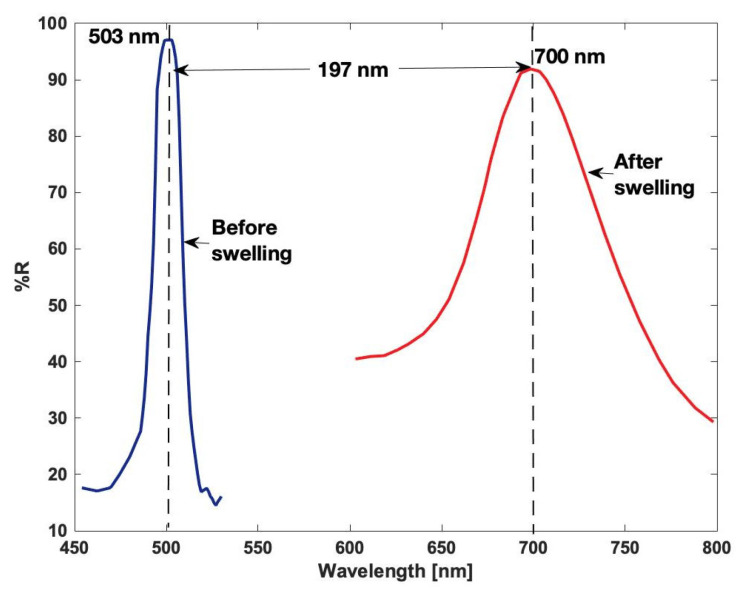
Behavior of a DCG mirror reflectance (%) as a function of wavelength (nm). The shift of the central wavelength is almost 200 nm [45]. Adapted from Markova, B., Nazarova, D., and Sharlandjiev, P., Control of the spectral position of dichromated gelatin reflection holograms. *Appl Opt*, 2011, 50, 5534–5537. Reproduced with permission of the publisher.

The initial spectral position is closely related to the wavelength of the recording laser, but it can be controlled by different factors that affect the final position [43]. For example, the swelling and shrinking occurring during the fabrication process of DCG holograms may influence the spectral position [44]. Applying well-thought treatments to the DCG holograms, shifts of up to 200 nm have been reported by Markova et al. in [45] (Figure 7). Some reports have mentioned a device wavelength tunability of around 1.2 and 1.6 μm [44]. The effects of filler materials have been reported in different works [45,46,47]. Specifically, the use of water-soluble filler materials during the fabrication process can shift shorter wavelengths of the UV or blue spectra [45]. In [46], blue shifts were also observed for higher gelatin-to-filler ratios. The oppositive, red shifting, was observed when the filler was included in the gelatin in a wet process, causing expansion of the spatial frequency. Due to the swelling of the gelatin during fabrication, the distance between fringes, d can be expanded, resulting in a longer reconstruction wavelength [47]. The spectral position also shows a dependence on the incidence angle; shifts of up to 50 nm have been demonstrated for a 50 degree variation in [48].

The control of the holograms’ reflection bandwidth can be achieved by controlling the manufacture and development processes, but it is long and complicated; thus, it cannot be described in this review. Reference [49] gives several recipes to achieve narrow or wide bandwidths.

As seen from Equation (1), changes in the refractive index can also modify the spectral position. These changes can be due to crosslinking or the addition of dye particles. Crosslinking refers to the interaction of light-sensitive elements with DCG, which may affect the refractive index and, therefore, the Bragg wavelength [46,48]. Crosslinking in DCG may also affect its mechanical and optical properties, allowing for a shift in the final spectral position. The crosslinking may be controlled with the processing parameters, allowing the tuning of the spectral position [48]. In the case of dye addition, dyes like methylene green and methylene blue enhance the spectral sensitivity of dichromated gelatin, extending its range to red wavelengths [47].

Besides the previously described applications of solar technologies, other applications for controlling the spectral position of DCG holograms are color holography [49] and optical filters [50].

## 9. DCG in Light Sources

In the beginning, the laser structures presented an active gain medium, usually a gas in a glass cylinder, and a feedback mechanism that usually were two high-reflectance mirrors; one of them let through light of weak intensity. However, new microstructures for lasing have appeared, and, in one of them, the mirrors have been replaced by icosahedral quasicrystals that were recorded in DCG plates [51]. The recording configuration to make the icosahedral prisms had seven coherent beams that formed the icosahedral pattern. Polarization of the beams was considered. After the recording step, the DCG plates were developed by wetting them in a mixture of water at 15 °C and 2.5 × 10^−4^ g/mL of organic dye Rhodamine 590 for 30 min. To study the emission spectrum of the dye-doped DCG icosahedral quasicrystals, they were illuminated with a Nd-YAG laser emitting at 532 nm, with 35 ps wide pulses and a 10 Hz repetition rate. For the spectral measurements, a CCD spectrometer that had a spectral range between 536 and 697 nm was used.

## 10. Optical Elements Made with DCG

**Figure 8:**
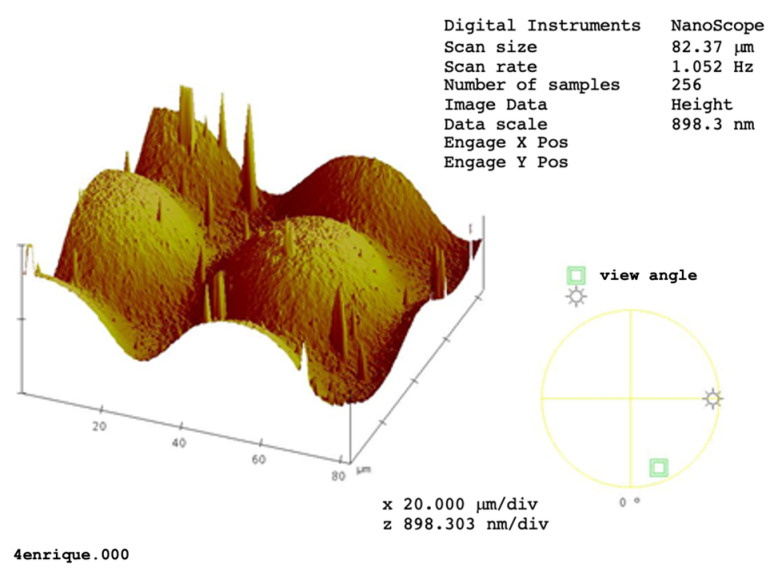
AFM image of part of a DCG lens array [52]. Reproduced from Navarrete-García, E., and Calixto, S., Continuous surface relief micro-optical elements fabricated on photographic emulsions by use of binary and halftone masks. *Optical Materials*, 2003, 23(3–4), 501–512. https://doi.org/10.1016/S0925-3467(03)00004-1. Reproduced with permission of the publisher.

DCG is mostly used to make optical elements recorded in the bulk of the DCG film, which is laid over a glass substrate. However, micro-optical elements in photographic plates can be fabricated showing a gelatin surface modulation like diffraction gratings, lenses, and mirrors [52]. To make microlenses, an image of a high-contrast mask that had circular holes was projected over a plate. Then, an exposure on the plate was given, and a development followed. The developed plate showed small surface lenses with diameters of about hundreds of µm (Figure 8). By using an interference microscope, the sagitta of the lenses was measured and resulted to be of about some micrometers. Focal distances were in the range of some millimeters. Negative-surface lenses can be coated with an aluminum thin film, and, in this way, they behave as mirrors. If light impinged from the gelatin side, the mirrors formed a real image, but, if the light impinged from the glass substrate side, the image was virtual. 

## 11. DCG Gratings Used in Hygrometers

The amount of moisture in the air is the humidity, which is measured with a hygrometer. This parameter affects our daily lives in activities like the automotive, food, meteorological, semiconductor, building and construction, and medical industries

**Figure 9:**
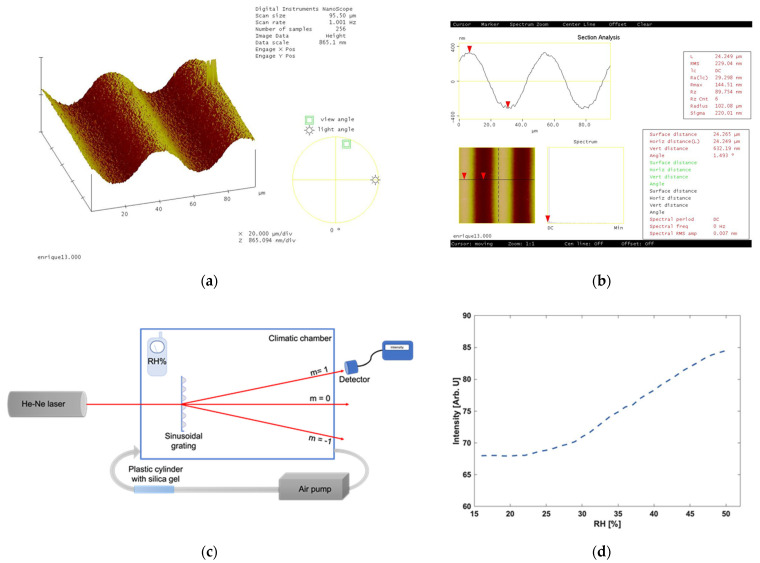
(**a**) AFM image of a surface sinusoidal grating made with gelatin [52]. (**b**) Profile of the grating shown in (**a**). (**c**) Set up used in the experimental step. Intensity of the first order (m = 1) was measured. A climate chamber was used to evacuate moisture. (**d**) Calibration plot relating first-order intensity versus relative humidity, as presented in [53]. Figure 9a Reproduced from Navarrete-García, E., and Calixto, S., Continuous surface relief micro-optical elements fabricated on photo-graphic emulsions by use of binary and halftone masks. *Optical Materials*, 2003, 23(3–4), 501–512. https://doi.org/10.1016/S0925-3467(03)00004-1. Reproduced with permission of the publisher.

New humidity sensors are needed for different applications. A material that is highly hygroscopic is gelatin. This characteristic is due to the high number of polar groups [1] in its molecular structure. Water molecules are absorbed and adsorbed by the gelatin films’ surface where there are physical and chemical interactions. Also, there are modifications in the bulk of the film due to water accumulation on and diffusion into the film. When gelatin absorbs water molecules, it swells, and, under desorption, it shrinks. Reference [53] shows a diffraction grating sensor. The gratings were made by recording on a DCG plate the interference pattern of two beams (Figure 9a,b). Gratings were placed in a climatic chamber in which it was possible to control the humidity. When the humidity increases, the gelatin film will swell, and the opposite, when the humidity decreases, gelatin shrinks. This changed the profile of the surface grating and, thus, the intensity of the diffracted orders. The first-order intensity was measured (Figure 9c). The calibration curves consisted of the intensity of the first order as a function of the change in relative humidity (Figure 9d).

## 12. Photopolymers

Although this article is dedicated to the DCG material, it is good mentioning, briefly, another photosensitive material that could be used instead of DCG and has been widely developed since about 1970, the photopolymers [54]. They can be classified as follows: photopolymerizable systems, photocrosslinking systems, and doped polymer systems. Also, polymers can be divided with respect to the dry or liquid state. Between the photopolymer systems, there are ones that require development, while others need self-processing, and lastly others show dichromated gelatin mimetics. Also, there is another division according to the polarization mechanism: single-monomer systems (linear polymerization, crosslinking polymerization), two-monomer systems (linear polymerization), and multicomponent crosslinking polymer systems. Among the systems requiring post-treatment are the PMMA–titanocene and the acrylamide (Table 3). Among the materials that present post-treatment are those based on oligouretane, oli-gocarbonate, and oligoether acrylates and multiacrylates (Table 4). Systems that can be developed with a thermal process contain acrylate, methacrylate, vinyl carbazole, a binder, radical precursors, solvents, and sensitizer (Table 5).

Among the photocrossslinkable polymers, there are the monomer–polymer, the metal ion–polymer, the polyvinyl alcohol–Cr (III), the polyacrylic acid–Cr(III), the polyvinylalcohol–Fe(II) sytems, and others.

More recently, new studies have been developed to improve the photopolymer mixtures [55]. These studies deal with the optimization of the monomer, the crosslinker, the sensitizers, and the binders and use of dopants like the inorganic–organic. Thus, the mixtures now present an improvement in the spatial frequency response, low shrinkage, dynamic range, stability of the recorded patterns, and environmental stability. In addition, the response of photopolymers to some chemicals has allowed the making of sensors. Finally, the improvement of photopolymers with organic–inorganic nanocomposites has been studied, which improves the optical response. In Table 6 are shown some of the characteristics of photopolymers made with acrylates, acrylamides, and Thiol-“X”.

## 13. Recent Applications of DCG Plates

DCG plates have been applied recently to make optical components, for example, holographic solar concentrators that can choose light with different wavelengths and, thus, present high efficiency [66]; ultraviolet–blue volume-phase holographic gratings [67]; and high-frequency relief-phase holographic gratings [68]. Novel applications such as distributed feedback lasers [69], direct laser writing [70], and others [71,72,73,74,75,76] have also been reported.

Some recent applications of volume holographic optical elements, made with DCG plates, have been implemented in 3D displays used in augmented and virtual realities. A waveguide eye-tracking system was reported in [77]. DCG has been mentioned as one of two promising materials for designing holographic lenses that couple light into AR eyewear substrates, making them suitable for eye motion-sensing applications [77,78]. A short review about optics for head-mounted displays, focusing on holographic and diffractive optics, can be found in [79].

A reference that also mentions the fabrication of relief optical components with DCG is Reference [80]. 

## 14. Conclusions

This review presents a general overview of the following: the components that comprise the DCG films, an explanation of the photochemical processes developed in the DCG layer; DCG films’ spectral sensitivity, fabrication, and developing processes; and characterizing methods. In addition, some applications have also been shown. However, because DCG films have been used through several decades, more information exists on the films, processing steps, and applications in the literature that give details.

## Figures and Tables

**Table 1:** Experimental results showing the dependence of diffraction efficiency and bandwidth with the concentration of the sensitizing mixture of water and dichromate. Thickness is the parameter [4] ^1^.

Concentration [%]	Thickness (μm)	Diffraction Efficiency [%]	Bandwidth [nm]
2	12.5	90	16
2	15.2	95	16
5	12.8	97	17
5	14.9	99.3	20
10	12.3	99.5	25
10	16.1	99.4	22

^1^ Reproduced from Pawluczyk, R., Billard, T. C., Quaglia, A., Vienneau, T., and Hockley, B. S. Characterization of DCG Holograms During the Production Process: Some Practical Aspects, in Proc. SPIE 0954, *Optical Testing and Metrology II*, (16 January 1989). Reproduced with permission of the publisher.

**Table 2:** Sensitivity of Dichromated Gelatin plates [8] ^1^.

Wavelength and Conditions	Thickness [μm]	Grating Period [μm]	Sensitivity [mJ/cm²]
448 nm	1	0.5	2.3
	3	0.5	1.7
	7	0.5	2.3
	10	0.5	1.5
	15	0.5	1.9
448 nm, 649F plates	12	1.4	6.6
	12	0.28	1.2
	12	0.16	1.6
448 nm, 649F plates	14	0.4–10	2.5
441 nm, 649F plates	13	~0.5	5

^1^ Adapted form Smith H. M. Ed., *Holographic Recording Materials*, Springer-Verlag: New York, USA, 1977, pp.75–99. Reproduced with permission of the publisher.

**Table 3:** Features of photopolymers that need development [54] ^1^.

Reference	Presentation	Film Thickness [μm]	Exposure Wavelength [nm]	Sensitivity [mJ/cm²]	Resolution [lines/mm]	Diffraction Efficiency [%]
PMMA– titanocene	PMMA block	500–3000	514	4000	—	~100

^1^ Original data published in Calixto, S., Lougnot, D., and Naydenova, I., Light-sensitive materials: Silver Halide Emulsions, Photoresist and Photopolymers. In *Handbook of Optical Engineering, 2nd ed*.; Malacara, D., Thompson, B.J., Marcel Dekker: New York, NY, 2001; Chapter 25. Reproduced with permission of the publisher.

**Table 4:** Features of photopolymer systems with self-processing properties [54] ^1^.

Reference	Presentation	Film Thickness [μm]	Exposure Wavelength [nm]	Sensitivity [mJ/cm²]	Resolution [lines/mm]	Diffraction Efficiency [%]
Diluent + oligomers (FPK-488)	Liquid between glass plates	20	300–500	20	1500–6000	80
Diluent + oligomers (FPK-488)	Liquid between glass plates	20	633	50	—	60
Pre-polymerized multicomponents (PHG###)	Liquid between glass plates	20–100	450–800	100–500	>3000	80

^1^ Original data published in Calixto, S., Lougnot, D., and Naydenova, I., Light-sensitive materials: Silver Halide Emulsions, Photoresist, and Photopolymers. In *Handbook of Optical Engineering, 2nd ed.*; Malacara, D., Thompson, B.J., Marcel Dekker: New York, NY, 2001; Chapter 25. Reproduced with permission of the publisher.

**Table 5:** Features of photopolymer systems involving the crosslinking of a polymer structure [54] ^1^.

Reference	Presentation	Film Thickness [μm]	Exposure Wavelength [nm]	Sensitivity [mJ/cm²]	Resolution [lines/mm]	Diffraction Efficiency [%]
*p*-Vinylcarbazole	Dry film on glass	2.5–7	488	50–500	800–2500	80
PMMA	Dry film on glass	100–200	488	7000	2000	~100
DCPVA	Dry film on glass	30–60	488	500	3000	~70
DCPAA	Dry film on glass	60	488	200	3000	~65
FePVA	Dry film on glass	60	488	>15,000	3000	80

^1^ Original data published in Calixto, S., Lougnot, D., and Naydenova, I., Light-sensitive materials: Silver Halide Emulsions, Photoresist, and Photopolymers. In *Handbook of Optical Engineering, 2nd ed*.; Malacara, D., Thompson, B.J., Marcel Dekker: New York, NY, 2001; Chapter 25. Reproduced with permission of the publisher.

**Table 6:** Features of volume holographic transmission gratings when they were recorded with different materials [55] ^1^.

Photopolymer	Reference	∆n_max_	Thickness [μm]	Wavelength [nm]	Spatial Frequency [lines/mm]
Acrylate	Shen et al. [56]	0.08	15	532	4949
Acrylate	Shen et al. [57]	0.065	12	532	N/A
Acrylate	Guo et al. [58]	0.046	5	633	3250
Acrylamide	Rogers et al. [59]	0.005	36	633	800
Acrylamide	Pi et al. [60]	N/A	140	532	N/A
Acrylamide	Zhang et al. [61]	0.034	10	633	1333
Thiol-‘X’	Hu et al. [62]	0.04	5–10	633	2000
Thiol-‘X’	Galli et al. [63,64]	0.0346	16.2	633	1200
Thiol-‘X’	Mavila et al. [65]	0.018	11	633	2500

^1^ Adapted from M. Murray, I. Naydenova, and S. Martin, Review of recent advances in photosensitive polymer materials and requirements for transmission diffractive optical elements for LED light sources, *Opt. Mater. Express*, 2023, 13, 3481–3501. Reproduced with permission of the publisher.

## Data Availability

No new data were created or analyzed in this study. Data sharing is not applicable to this article.
